# Acidic Electrolyzed Water Inhibits the Viability of *Gardnerella* spp. *via* Oxidative Stress Response

**DOI:** 10.3389/fmed.2022.817957

**Published:** 2022-02-25

**Authors:** Chongyu Zhao, Yu Chen, Lvfen Gao, Jue Huang, Xiurou Yang, Luowei Pei, Zhangying Ye, Linyan Zhu

**Affiliations:** ^1^Department of Pharmacology, School of Medicine, Jinan University, Guangzhou, China; ^2^Department of Urology, The Third Affiliated Hospital, Sun Yat-sen University, Guangzhou, China; ^3^Department of Obstetrics and Gynecology, The First Affiliated Hospital, Jinan University, Guangzhou, China; ^4^School of Biosystems Engineering and Food Science, Zhejiang University, Hangzhou, China

**Keywords:** acidic electrolyzed water, *Lactobacillus acidophilus*, reactive oxygen species, *Gardnerella* spp., bacterial vaginosis (BV)

## Abstract

The vaginal microbiota, dominated by Lactobacilli, plays an important role in maintaining women's health. Disturbance of the vaginal microbiota allows infection by various pathogens such as *Gardnerella* spp. (GS) and related anaerobic bacteria resulting in bacterial vaginosis (BV). At present, the treatment options for BV are extremely limited. Treatment of antibacterial drugs and vaginal acidification are the two primary therapeutic methods. Acid electrolyzed water (AEW) is known to inactivate microorganisms and is considered a medical application in recent years. Studies have found that *Lactobacillus acidophilus* (LA) probiotics helps to inhibit GS-induced BV. Our study took GS and LA as the research object, which aims to explore AEW as a potential alternative therapy for BV and its underlying mechanisms. We first obtained the pH of AEW (3.71–4.22) close to normal vaginal pH (3.8–4.5) to maintain normal vaginal acidification conditions. Plate counting experiments showed that AEW (pH: 4.07, ORP: 890.67, ACC: 20 ppm) (20 ppm) could better inhibit the viability of GS but had a more negligible effect on LA. Then, we preliminarily explored the possible mechanism of AEW anti-GS using cell biology experiments and transmission electron microscopy. Results showed that the membrane permeability was significantly increased and the integrity of cell membrane was destroyed by AEW in GS than those in LA. AEW also caused protein leakage and cell lysis in GS without affecting LA. Meanwhile, AEW induced a number of reactive oxygen species (ROS) production in GS, with no obvious LA changes. Finally, we found that 20 ppm AEW exhibited excellent antibacterial effect on the vaginal secretions of women diagnosed with BV by Amsel criteria and sialic acid plum method. Taken together, our findings manifest that 20 ppm AEW has an excellent antibacterial effect in GS with less effect on LA, which might be expected to become a potential therapy for BV.

## Introduction

Bacterial vaginosis (BV) is manifested as various symptoms of mucosal inflammation, which include abnormal vaginal, itching, and burning, and those have been associated with a wide array of health issues such as preterm births, pelvic inflammatory disease, increased susceptibility to human immunodeficiency virus (HIV) infection, and other chronic health problems (1). BV represents a profound shift in the vaginal microbiota from the dominant Lactobacilli to a polymicrobial microbiota. Under physiological conditions, the vaginal microbiota is dominated by Lactobacilli, which maintains the acidic pH environment of the vagina (3.8–4.5) and release H_2_O_2_ to inhibit other bacteria growth (2). Instead, *Gardnerella* spp. (GS) and some other anaerobic bacteria multiply and eventually leading to BV (3).

The standard of care for BV treatment recommended from the Center for Disease Control (CDC) as metronidazole (MNZ) or clindamycin (4) has failed to control the recurrence of BV (more than 30% recurrence of BV on microscopy within 6 months) (5). Besides, they are both associated with various side effects such as gastrointestinal upset (6). Various antibacterial agents have been used to treat BV in recent years, such as chlorhexidine, hydrogen peroxide, polyhexamethylene biguanide, etc. (7). However, recent systematic reviews have confirmed that most studies on bactericides for BV are weak in methodology and that subsequent studies have been minimal (8). In addition, the use of these antibacterial drugs is mostly for widespread sterilization or the symptoms of patients, rather than only for pathogens. Their safety in the treatment of BV is questionable (9). This also indicates that designing a tailor-made drug that only targets pathogens is significant. The use of plant-derived compounds in the treatment of genital infections is increasing (10). A plant-derived compound called Lifukang lotion is used in China to treat BV. Currently, both practitioners and patients have widely recognized the limitations of successful BV treatment, and treatment options are still extremely limited. Novel therapies of BV are not likely to be available in the immediate future. Hence, reliance on the optimal use of available agents still is essential (11).

Acid electrolyzed water (AEW) is an environmentally friendly, broad-spectrum microbial disinfectant (12). Its principle is produced by electrolyzing a dilute sodium chloride or hydrochloric acid solution in an electrolytic cell without a separation membrane. The germicidal ingredients of AEW are available chlorine compounds, which include ClO^−^, HClO, and Cl_2_ (13). A schematic of the AEW generator is shown in Supplementary Figure S1. AEW features include high antibacterial efficiency and low available chlorine concentration (ACC). The reduction of chlorine will minimize the potential harm to human health and the environment (14). AEW has been widely used in the food disinfection industry. At present, AEW has begun to be considered explored in the medical fields, such as promoting skin healing and eliminating the COVID-19 (15, 16). According to the antibacterial effect and pH characteristics of AEW, we conjecture that AEW has good application potential for the treatment of BV.

Studies have found that *L. crispatus* and *L. jenseni* are the most commonly detected Lactobacilli in the vagina of healthy women (17). However, the number of Lactobacilli in BV-positive patients will drop sharply or disappear (18). *Lactobacillus acidophilus* (LA) is a type of vaginal Lactobacilli. An exciting experiment has found that treating BV with a vaginal douche containing a strain of LA contributed to help the regrowth of other Lactobacilli (19). Another study showed that LA is the most effective at attenuating BV induced by GS, followed by *Lactobacillus rhamnosus* (20). We aim at the BV treatment and envisage BV treatment with AEW containing LA strains, but this must take into account the antibacterial effect of AEW to LA.

In this study, we investigated the antibacterial potential of AEW (compared with MNZ and Lifukang) against GS, LA, and microorganisms found in vaginal secretions of BV patients. In addition, we also preliminarily investigated the anti-GS possible mechanism of AEW by cell biology experiments and transmission electron microscopy.

## Methods and Materials

### Preparation and Physicochemical Property Measurements of AEW, Lifukang Lotion, and MNZ

Acid electrolyzed water was produced by electrolyzing an electrolyte containing 6% HCl using a flow electrolyzer (HD-240L, Fuqiang-Want Sanitary Accessories Ltd, Shanghai, China). It was prepared on the experiment day and used within 20 min after production. The physicochemical properties of AEW were measured immediately after production. pH and ORP of AEW were measured by pH Meter (PHS-3CW, Shanghai Leigu Instrument Co., Ltd., China) and ORP Meter (SX712, Sanxin International Electric Shanghai Co., Ltd., China). Moreover, ACC was assessed by standard iodometric titration (21). For Lifukang lotion (Xi'an Taiji Pharmaceutical Co., Ltd.), it was diluted with distilled water to a concentration of 10% according to its instructions. Metronidazole (MNZ) tablets (Guangdong Huanan Pharmaceutical Group Co., Ltd., China) were dissolved in normal saline to prepare a 0.75% (m/m) metronidazole solution.

### Strains and Culture Conditions

*Lactobacillus acidophilus* ATCC 4356 and *Gardnerella* spp. (GS) ATCC 49145 were both obtained from Guangdong Microbial Culture Collection Center, China. LA was recovered by transferring to MRS Broth (Huankai Microbial Sci. & Tech. Co., Ltd., Guangdong, China) at 37°C in a 5% CO_2_ atmosphere for 48 h and stored in MRS Broth containing glycerin (volume ratio of 7:3) at −80°C. GS was recovered at Anaerobic Blood Agar Plate (Huankai Microbial Sci. & Tech. Co., Ltd., Guangdong, China) for 48 h in an anaerobic incubator (MGC, AnaeroPack C35, and MGC AnaeroPack C32, MITSUBISHI GAS CHEMICAL CO., INC., Japan) and stored in BHI Broth (Huankai Microbial Sci. & Tech. Co., Ltd., Guangdong, China) containing glycerin (volume ratio of 7:3) at −80°C.

### Preparation of Bacterial Suspension

The preparation of the bacterial suspension referred to our previous experiment (22). Before each experiment, scraped the *Gardnerella* spp. cultured at 37°C for 48 h on the anaerobic blood agar plate (Huankai Microbial Sci. & Tech. Co., Ltd., Guangdong, China) and pooled them into 5 ml sterile distilled water. LA was incubated (MRS medium, Huankai Microbial Sci. & Tech. Co., Ltd., Guangdong, China) in an incubator shaker (SHA-CA, Honghua Instrument Factory, Jintan, China) for 48 h at 150 r/min, 37°C. The above 5 ml GS and LA enriched cultures were collected into a sterile centrifuge tube and let stand for 10 min at 5,000 rpm and 4°C in a refrigerated centrifuge (5424 R, Eppendorf China Ltd., Shanghai, China). The precipitate was collected and washed two times with sterile distilled water, resuspended in sterile distilled water to obtain a final cell concentration of ~10^8^ CFU/mL, which was confirmed by plating 1-mL portions of appropriately diluted suspension on plate count agar plates and then incubated at 37°C for 48 h.

### Effect of AEW, MNZ, and Lifukang on Microbiota Inactivation

The antibacterial test of AEW was carried out concerning the method described previously (23, 24). In short, 1 mL of bacterial solution and 9 mL of treatment solution (AEW, MNZ, Lifukang) were incubated at 37°C for 5 min, 10 min, and 20 min, respectively. After the treatment for a predetermined period, the samples were mixed with the neutralizing buffer solution (0.5% Na_2_S_2_O_3_) at a ratio of 1:9 (1 ml:9 ml) for 5 min to neutralize the effect of the AEW. For Lifukang and MNZ, centrifuged (at 5,000 rpm, 4°C for 10 min) (5424 R, Eppendorf China Ltd., Shanghai, China) and sterile distilled water washed cells once to stop their action. In addition, samples were serially diluted in sterilized distilled water. The plates (GS: Anaerobic Blood Agar Plate, LA: MRS Plate) were incubated at 37°C for 48 h. Finally, the number of colonies developed on the plates was determined, and the results were expressed as log10 CFU/mL (Supplementary Figure S2).

### Propidium Iodide Staining

The solution used in this part of the experiment and its concentration refer to our previous experiment (22). AEW and cell suspensions (same as Section Preparation of bacterial suspension) were mixed at a volume ratio of 9:1 (9 ml:1 ml) for 20 min at 37°C. The samples were mixed with the neutralizing buffer solution (0.5% Na_2_S_2_O_3_) at a ratio of 1:9 (1 ml:9 ml) for 5 min to neutralize the action of the AEW. Washed the cells once, then added 100 μL PI (Propidium iodide) diluent (Beyotime Biotechnology, China) (100 μL binding buffer + 2.5 μL PI) to each sample and mixed well. Then, the specimens were left to stand in the dark for 10 min. Quantitative measurement of PI stain was performed by flow cytometry, and qualitative analysis of PI stain was observed under fluorescence microscopy.

Flow cytometry: NovoExpress software was used to observe the sample results and set the number of samples. Since most GS was destroyed by 20 ppm AEW, after many items of washing and centrifugation, only 5^*^10^4^ bacteria were finally collected. The treated cells were examined by a BC flow cytometer (GALLIOS, BECKMAN COULTER, USA) (488 nm excitation and 630 nm emission). The relative fluorescence intensities of this experiment were calculated as follows: (fluorescence intensity of the experimental group/fluorescence intensity of the control group) × 100%.

Fluorescence microscopy (IX71, Olympus, Japan): dropped one drop of the above-stained cell suspension onto a glass slide and covered the cells with a coverslip. Under the fluorescence microscope, the PI fluorescence signal is red (510–560 nm excitation and over 590 nm emission).

### Hoechst Staining

Acid electrolyzed water and cell suspensions (same as Section Preparation of bacterial suspension) were mixed at a volume ratio of 9:1 (9 ml:1 ml) for 20 min at 37°C. Then, the samples were mixed with the neutralizing buffer solution (0.5% Na_2_S_2_O_3_) at a ratio of 1:9 (1 ml:9 ml) for 5 min to neutralize the effect of the AEW. After centrifugation (at 5,000 rpm, 4°C for 10 min) (5424 R, Eppendorf China Ltd., Shanghai, China), the supernatant was discarded and the cells were washed once with sterile distilled water. They were then resuspended. Cell suspensions were added with 1 ml Hoechst 33258 (Beyotime Biotechnology, China) (10 μg/mL). We placed the samples for 15 min at room temperature in the dark. Then, the cells were washed once and resuspended in sterile distilled water. Finally, cell suspensions were observed with a fluorescence microscope within 20 min.

### BCA Protein Assay

Acid electrolyzed water and cell suspensions (same as Section Preparation of bacterial suspension) were mixed at a volume ratio of 9:1 (9 ml:1 ml) for 20 min at 37°C. The samples were mixed with the neutralizing buffer solution (0.5% Na_2_S_2_O_3_) at a ratio of 1:9 (1 ml:9 ml) for 5 min to neutralize the effect of the AEW. After centrifugation (at 5,000 rpm, 4°C for 10 min) (5424 R, Eppendorf China Ltd., Shanghai, China) was completed, the supernatant of each sample was taken as a sample to be tested. BCA Protein Assay (BCA) Kit was obtained from Beyotime, China. According to the instructions of the kit, 20 μl of the supernatant was mixed with 200 μl of the working solution in a 96-well plate according to the kit's instructions, and they were incubated at 37°C for 20 min. The samples were then tested with a microplate reader (570 nm). The protein content of the supernatant was calculated from the standard curve.

### Transmission Electron Microscopy

Acid electrolyzed water and cell suspensions (same as Section Preparation of bacterial suspension) were mixed at a volume ratio of 9:1 (9 ml:1 ml) for 20 min at 37°C. The samples were mixed with the neutralizing buffer solution (0.5% Na_2_S_2_O_3_) at a ratio of 1:9 (1 ml:9 ml) for 5 min to neutralize the effect of the AEW. The cells were washed once with sterile distilled water and the supernatant was removed. Cell pellets were fixed with 2.5% neutral glutaraldehyde (2.5% neutral glutaraldehyde with 0.1 M phosphate buffer) (manufactured by Ala Aesar, A17876) for 2 h. The cells were washed with 0.1 M phosphate buffer (Sinopharm Group Co., Ltd.) 6 times, 30 min each time. The cells were fixed with 1% osmium acid (TED PELLA, 18456) for 2 h. The cells were rinsed with 0.1 M phosphate buffer (Sinopharm Group Co., Ltd.) 3 times for 10 min each. A total of 50–100% ethanol (Sinopharm Group Co., Ltd.) was used for dehydration, 10 min each time. 100% acetone (Sinopharm Group Co., Ltd., 191105-1) and 100% ethanol (Sinopharm Group Co., Ltd.) was mixed 1:1 to prepare a solution which acted on the cells for 15 min. 100% acetone (Sinopharm Group Co., Ltd., 191105-1: Epon812) resin (TED PELLA, GP18010) was mixed with 1:2 solution for infiltration and embedding. Ultra-Microtome (UC-7, Leica, Germany) was used for the resin-embedded samples which were sliced serially and stained with 2% uranyl acetate (EMS, 22400) for 30 min and lead citrate (TED PELLA, 19314) for 15 min. Finally, the prepared sample was observed by a transmission electron microscope (Nippon Electron Optics Laboratory Co., Ltd., JEM-1400 PLUS). Used voltage: 100 KV, found the slice in the phosphor screen at low magnification, inserted CCD (camera model: VELETA G3; brand: EMSIS; acquisition and measurement software: RADIUS) to observe the target structure and debug electron microscope until the image was clear, collected the required image.

### Reactive Oxygen Species Measurement

Reactive oxygen species (ROS) detection kit (Beyotime Biotechnology, China, China) was used for the detection of the cell's ROS levels by fluorescence probe (DCFH-DA, (2,7-dichlorodi-hydrofluorescein diacetate). AEW and cell suspensions (same as Section Preparation of bacterial suspension) were mixed at a volume ratio of 9:1 (9 ml:1 ml) for 20 min at 37°C. The samples were mixed with the neutralizing buffer solution (0.5% Na_2_S_2_O_3_) at a ratio of 1:9 (1 ml:9 ml) for 5 min to neutralize the effect of the AEW. After centrifugation (at 5,000 rpm, 4°C for 10 min) (5424 R, Eppendorf China Ltd., Shanghai, China), the supernatant was discarded and the cells were washed with sterile distilled water once. They were then resuspended. Cell suspensions were then washed once and resuspended in 200 μL of LDCFH-DA working solution and incubated for 20 min at 37°C in the dark. Quantitative and qualitative experiments were performed using flow cytometry and fluorescence microscopy.

Fluorescence microscopy (IX71, Olympus, Japan): dropped one drop of the above-stained cell suspension onto a glass slide and covered the cells with a coverslip. Under the fluorescence microscope, the FDA fluorescence signal is green (450–490 nm excitation and over 520 nm emission).

Flow cytometry: the ROS content was measured by a BC flow cytometer (488 nm excitation and 525 nm emission). NovoExpress software was used to observe the sample results and set the number of samples to 5^*^10^4^. Since most GS was destroyed by 20 ppm AEW, after many items of washing and centrifugation, only 5^*^10^4^ bacteria were finally collected. The relative fluorescence intensities of this experiment were calculated as follows: (fluorescence intensity of the experimental group/fluorescence intensity of the control group) × 100%.

### Collection and Processing of BV Specimens

Vaginal secretion samples were from BV patients treated in the First Affiliated Hospital of Jinan University from July 26, 2019, to December 31, 2019. Vaginal swabs from BV patients were obtained from the posterior fornix. The diagnosis of BV was based on the existence of at least three of the four Amsel criteria (25): 1. vaginal pH was >4.5; 2. the presence of clue cells (>20%); 3. milky, homogenous vaginal discharge; and 4. amine (fishy) odor after adding 10% KOH to the vaginal fluid. All vaginal secretion samples were tested positive by the laboratory of the First Affiliated Hospital of Jinan University using the sialic acid plum method. In addition, we assessed the presence or absence of Candida and Trichomonas vaginalis using electron microscopy. A history of BV recurrence was defined as having at least two acute attacks in the past 12 months. Non-recurrent BV patients had symptoms of BV for the first time and had not received any treatment.

The secretions were stored in sterile distilled water and processed within 1 h. A spectrophotometer (OD570) was used to record the absorbance of each sample and adjusted to ensure that the absorbance of each sample was the same. All drugs (AEW, Lifukang, and MNZ) and cell suspensions were mixed at a volume ratio of 9:1 (9 ml:1 ml) for 20 min at 37°C. The samples were mixed with the neutralizing buffer solution (0.5% Na_2_S_2_O_3_) at a ratio of 1:9 (1 ml:9 ml) for 5 min to neutralize the effect of the AEW. After centrifugation (at 5,000 rpm, 4°C for 10 min) (5424 R, Eppendorf China Ltd., Shanghai, China), the supernatant was discarded and the cells were washed in sterile distilled water once. They were then resuspended. Bacterial suspensions were smeared on the anaerobic blood agar plate (Huankai Microbial Sci. & Tech. Co., Ltd., Guangdong, China) for 48 h at 37°C in an anaerobic environment. The institutional ethics review committees of the hospital approved this analysis. The research was conducted following the Declaration of Helsinki. All patient data were anonymized in the paper.

### Statistics

We used Fisher's test to compare the antibacterial efficacy of drugs on BV samples. Other data were presented as mean ± standard error, analyzed by Student's *t*-test and ANOVA by SPSS 13.0. The statistical significance was indicated by a *p* < 0.05.

## Results

### Obtained Low pH, Different Concentrations of AEW

One of the essential treatments for BV is vaginal acidification. Some vaginal acidifiers, such as lactic acid gel, have been proven to treat and prevent BV (26). Based on this, we also took the maintenance and restoration of the vaginal microenvironment as the starting point and screened the pH of the AEW between 3.71 and 4.22 (normal vaginal pH: 3.8–4.5) (Table 1). At the same time, we also controlled the ppm value to a low concentration of 10 to 80 for reducing residual chlorine (Table 1).

**Table 1:** Properties of AEW with different ppm, Lifukang, and MNZ.

**Solutions**	**ACC^1^ (ppm)**	**pH**	**ORP^2^ (mV)**
Tap water	1.18 ± 0.33^a^	6.96 ± 0.02^a^	660.67 ± 29.95^a^
	10.97 ± 0.29^b^	4.22 ± 0.01^b^	843.67 ± 20.29^b^
	20.77 ± 0.33^c^	4.07 ± 0.03^c^	890.67 ± 6.13^c^
	30.44 ± 0.50^d^	3.85 ± 0.01^d^	893.00 ± 2.16^c^
	40.01 ± 0.76^e^	3.79 ± 0.02^d, f^	893.67 ± 20.89^d, c^
AEW^3^	50.98 ± 0.76^f^	3.74 ± 0.06^f^	901.67 ± 17.59^d, c^
	59.47 ± 0.58^g^	3.70 ± 0.03^f^	908.00 ± 29.41^d, c^
	69.38 ± 0.50^h^	3.71 ± 0.03^f^	968.67 ± 40.01^e, d^
	80.59 ± 1.09^i^	3.71 ± 0.03^f^	994.33 ± 33.81^f, e^
Lifukang (10%)	0^j^	5.29 ± 0.01^i^	234.00 ± 4.32^f, e^
MNZ^4^ (0.75%)	0^j^	5.67 ± 0.02^j^	243.67 ± 2.87^f, e^

1 *ACC, available chlorine concentration (ppm)*.

2 *ORP, Oxidation reduction potential (mV)*.

3 *AEW, Acidic electrolyzed water*.

4 *MNZ, metronidazole*.

a−j *Values within the same column with different lowercase letters indicate significantly different (P < 0.05). Values represent the mean ± standard deviation (n = 3)*.

### Twenty ppm AEW Significantly Inhibited the Viability of GS, but Had Less Effect on LA

After determining the pH of AEW, we explored the antibacterial effect of different ppm values of AEW on GS and LA. As shown in Table 2, AEW (10 ppm) showed an obvious antibacterial effect on GS (reduction value was 0.22–0.43 Log CFU/mL). It was worth noting that when ACC was equal to or higher than 20 ppm, GS hardly grew on plates. Lifukang, a typically used rinsing agent in BV, showed a similar anti-GS effect as AEW (≥20 ppm). However, the anti-GS effect of MNZ was not as well as Lifukang and AEW. The reductions of GS populations by MNZ were only 1.27, 1.61, and 1.06 Log CFU/mL at 5, 10, and 20 min, respectively (Table 2).

**Table 2:** Treatment of AEW, Lifukang, and MNZ in GS.

**Treating solution**		**Treating time (mins)**					
		**5**		**10**		**20**	
		**Log Survival (CFU/mL) ±standard deviation (SD)**	**Log reduction (CFU/mL)**	**Log survival (CFU/mL) ±standard deviation (SD)**	**Log reduction (CFU/mL)**	**Log survival (CFU/mL) ±standard deviation (SD)**	**Log reduction (CFU/mL)**
Control		8.25 ± 0.04^a, A^		8.52 ± 0.27^a, A, B^		8.03 ± 0.07^a, A, C^	
	10	8.03 ± 0.14^a, A^	0.22	8.12 ± 0.06^b, A, B^	0.4	7.60 ± 0.03^b, A, C^	0.43
	20	ND^1^	8.25	ND	8.52	ND	8.03
	30	ND	8.25	ND	8.52	ND	8.03
	40	ND	8.25	ND	8.52	ND	8.03
AEW^2^ (ppm)	50	ND	8.25	ND	8.52	ND	8.03
	60	ND	8.25	ND	8.52	ND	8.03
	70	ND	8.25	ND	8.52	ND	8.03
	80	ND	8.25	ND	8.52	ND	8.03
Lifukang (10%)		ND	8.25	ND	8.52	ND	8.03
MNZ^3^ (0.75%)		6.98 ± 0.15^b, A^	1.27	6.91 ± 0.09^b, A^	1.61	6.97 ± 0.11^c, A^	1.06

1 *Not detected*.

2 *AEW, Acidic electrolyzed water*.

3 *MNZ, metronidazole*.

a−c *Values within the same column with different lowercase letters indicate significantly different (p < 0.05)*.

A−C *Values within the same row with different uppercase letters indicate significantly different (p < 0.05). Values represent the mean ± standard deviation (n = 3)*.

Subsequently, we elucidated the influence of AEW on LA. In Table 3, we could detect that AEW (10 ppm) exhibited a feeble antibacterial effect against LA at all periods (reduction value was −0.05 to 0.05 log CFU/mL). AEW showed a weak inactivation to LA microbiota when it reached 20 ppm (reduction value was −0.1, 0.18, and 0.19 log CFU/mL at 5, 10, and 20 min, respectively) (Table 3). However, the inhibition rate in LA (−27.08%, 32.56%, and 29.99% at 5, 10, and 20 min, respectively) was remarkably more diminutive than that in GS (almost 100% in all treatment time) (Supplementary Figure S3). Further, an increase in ACC to above 30 ppm led to apparent inactivation to LA (Table 3).

**Table 3:** Treatment of AEW, Lifukang, and MNZ in LA.

**Treating solution**		**Treating time (mins)**					
		**5**		**10**		**20**	
		**Log Survival (CFU/mL) ±standard deviation (SD)**	**Log reduction (CFU/mL)**	**Log survival (CFU/mL) ±standard deviation (SD)**	**Log reduction (CFU/mL)**	**Log survival (CFU/mL) ±standard deviation (SD)**	**Log reduction (CFU/mL)**
Control		7.71 ± 0.05^a, A^		7.71 ± 0.05^a, A^		7.75 ± 0.04^a, A^	
AEW^2^ (ppm)	10	7.72 ± 0.07^a, A^	−0.01	7.67 ± 0.05^b, a, A^	0.05	7.80 ± 0.10^b, A^	−0.05
	20	7.81 ± 0.11^a, A^	−0.10	7.53 ± 0.09^c, b, A^	0.18	7.56 ± 0.10^c, a, A^	0.19
	30	6.79 ± 0.17^b, A^	0.92	6.82 ± 0.09^d, A^	0.89	6.90 ± 0.16^d, A^	0.85
	40	5.66 ± 0.06^c, A^	2.05	5.67 ± 0.05^e, A^	2.04	5.97 ± 0.11^e, B^	1.78
	50	5.82 ± 0.08^d, A^	1.89	5.85 ± 0.13^f, e, A^	1.86	5.89 ± 0.09^f, e, A^	1.86
	60	4.00 ± 0.00^e, A^	3.71	4.05 ± 0.07^h, A^	3.66	4.56 ± 0.07^i, B^	3.20
	70	ND^1^	7.71	ND	7.71	ND	7.75
	80	ND	7.71	ND	7.71	ND	7.75
Lifukang (10%)		7.28 ± 0.06^f, b, A^	0.42	7.31 ± 0.06^i, b, A^	0.40	7.19 ± 0.05^j, d, A^	0.56
MNZ^3^ (0.75%)		7.49 ± 0.19^i, a, f, A^	0.22	7.45 ± 0.08^j, c, i, A^	0.26	7.34 ± 0.12^k, j, c, A^	0.41

1 *Not detected*.

2 *AEW, Acidic electrolyzed water*.

3 *MNZ, metronidazole*.

a−k *Values within the same column with different lowercase letters indicate significantly different (p < 0.05)*.

A, B *Values within the same row with different uppercase letters indicate significantly different (p < 0.05). Values represent the mean ± standard deviation (n = 3)*.

In consideration of Lifukang and MNZ, both displayed weak influence on LA, with the value of LA reduction being between 0.40–0.56 log CFU/mL and 0.22–0.41 Log CFU/mL, respectively (Table 3). It was worth mentioning that MNZ (62.43%, 59.47%, and 61.70% at 5, 10, and 20 min, respectively) and Lifukang (33.28, 49.67, and 59.66% at 5, 10, and 20 min, respectively) had higher inactivation to LA than AEW (20 ppm) (−27.08, 32.56, and 29.99% at 5, 10, and 20 min, respectively (Supplementary Figure S3).

These results indicated that AEW (20 ppm) could better inhibit the viability of GS and had a negligible effect on LA, which was better than Lifukang and MNZ. Based on these results, we selected AEW (20 ppm, 20 min) in the subsequent experiments.

### Twenty ppm AEW Increased the Permeability of GS With Less Effect on LA

**Figure 1:**
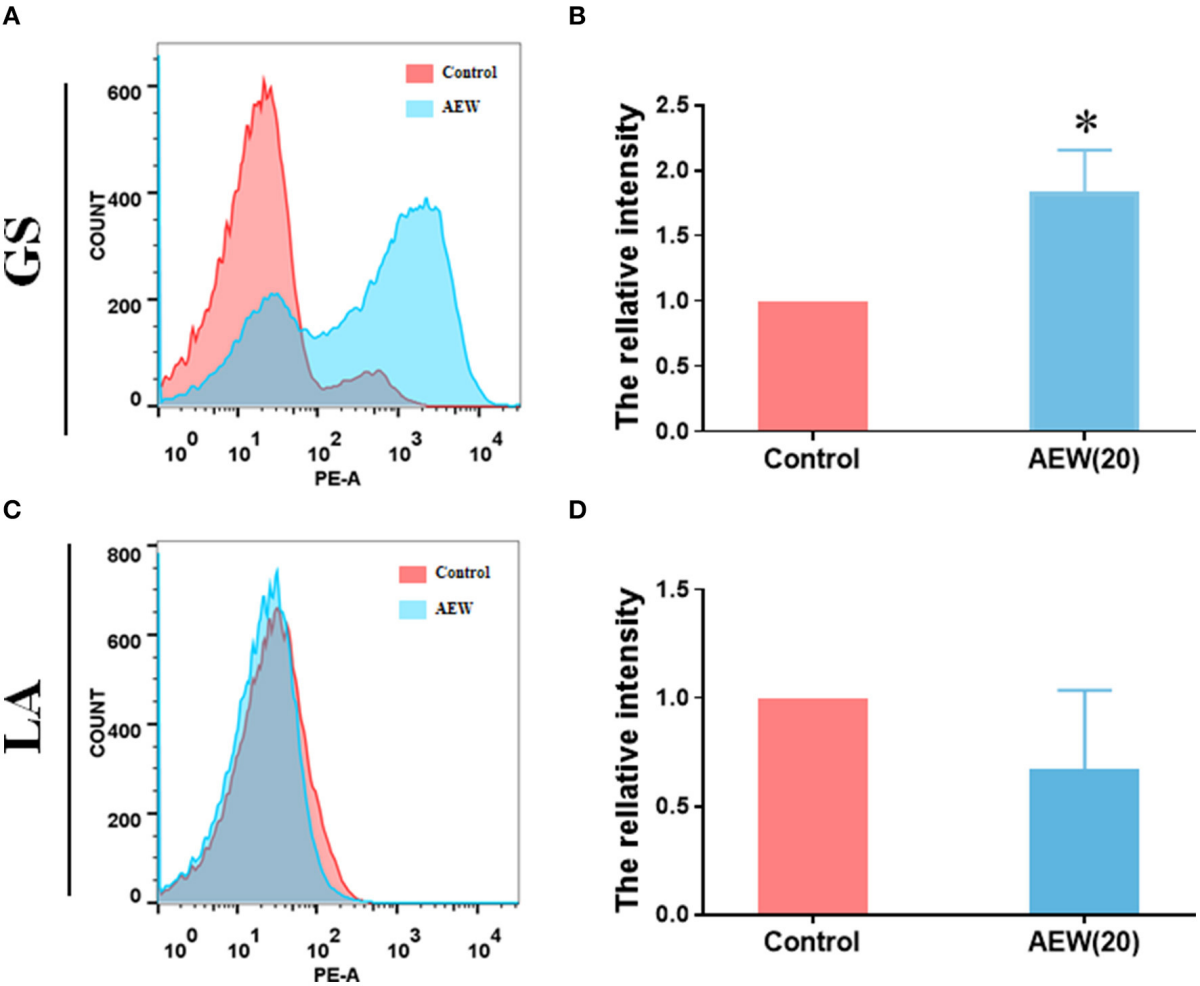
Twenty ppm AEW increased the permeability of GS with less effect on LA. **(A,C)** Representative cell permeability data were observed by flow cytometry. **(B,D)** Statistics of relative PI fluorescence intensity of GS and LA were measured by flow cytometry (*n* = 3, *p < 0.05 to control).

The results led to several questions about how does 20 ppm AEW kill GS, and what is the reason for the different inactivation effects of AEW on GS and LA. The primary antibacterial substance of AEW is the HOCl (27), and the cell membrane controls the entry and exit of small molecules. We first observed the changes in the cell membrane. PI is a nuclear dye that stains necrotic cells and cells with increased permeability (28). Flow cytometry and PI relative fluorescence intensity calculations revealed these results. We found that the relative PI average fluorescence intensity of GS treatment with AEW was higher than that in the control group (Figures 1A,B, *n* = 3, ^*^p < 0.05), whereas no notable difference could be observed in the LA group (Figures 1C,D, *n* = 3, ^*^p < 0.05). The qualitative experiments of PI and Hoechst under the fluorescence microscope also proved that after AEW treatment, the permeability of most GS increased and could be stained by PI and Hoechst (Supplementary Figure S4).

### Twenty ppm AEW Induced Structural Damage to GS but Had Less Effect on LA

**Figure 2:**
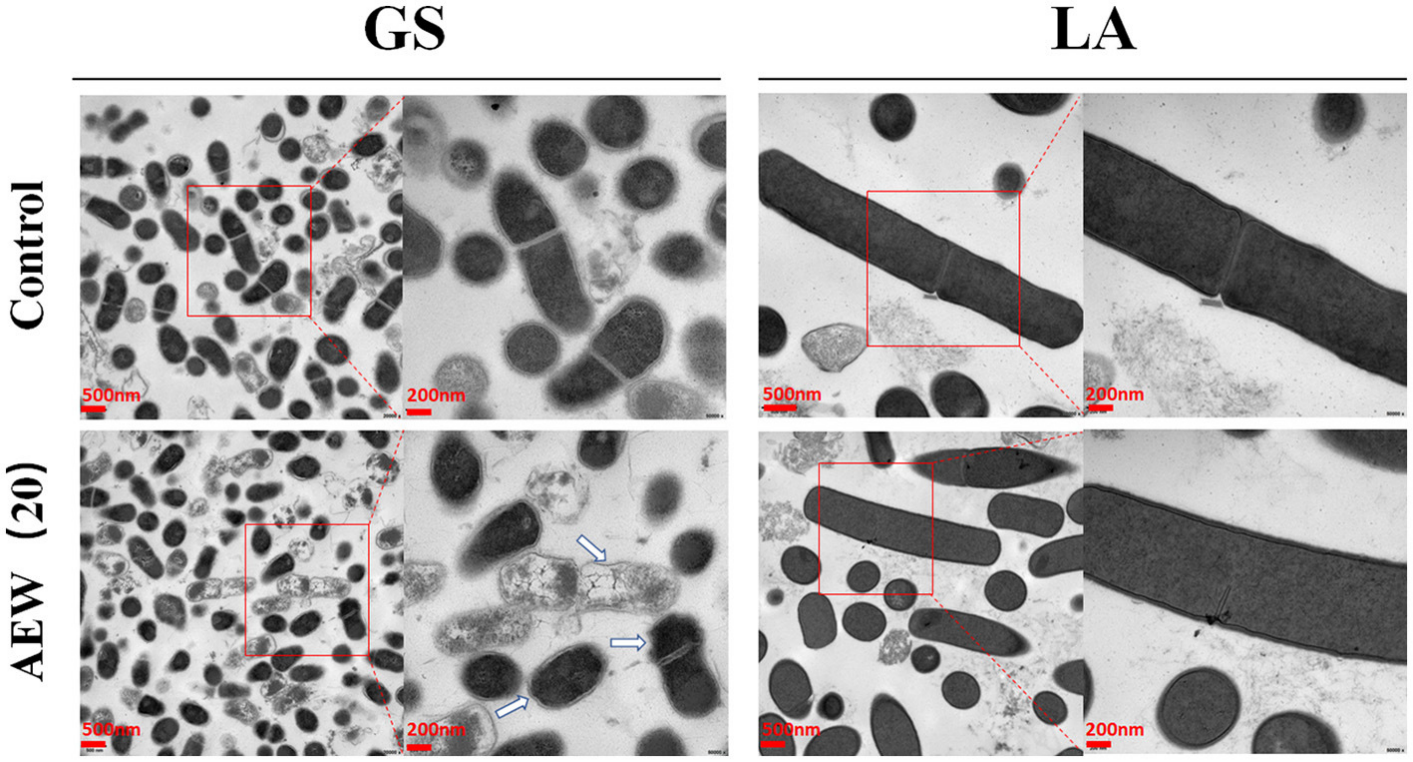
Twenty ppm AEW induced structural damage to GS but had less effect on LA. The image showed the internal structure of GS and LA observed under a transmission electron microscope after 20 ppm AEW treatment. The arrow indicated the destruction of the inside of the bacteria.

Transmission electron microscopy (TEM) was used to further analyze the changes in the internal structure of bacteria after AEW treatment. Here, we found that the GS control group was short rod-shaped, with continuous, smooth cell walls and cell membranes. Its cytoplasmic density was uniform and consistent. However, in the 20 ppm AEW treatment group, GS's cell membrane and cell wall had apparent wrinkles. It was worth noting that the cytoplasm of some GS was manifested as a large amount of cytoplasmic loss. On the contrary, regardless of whether it was the control group or AEW (20 ppm) treatment group of LA, they both had long rod-shaped, with continuous and smooth cell walls and cell membranes. Also, their cytoplasm density was uniform, without reduction and enhancement (Figure 2).

### Twenty ppm AEW Promoted Protein Leakage and Cell Lysis of GS With Less Effect on LA

**Figure 3:**
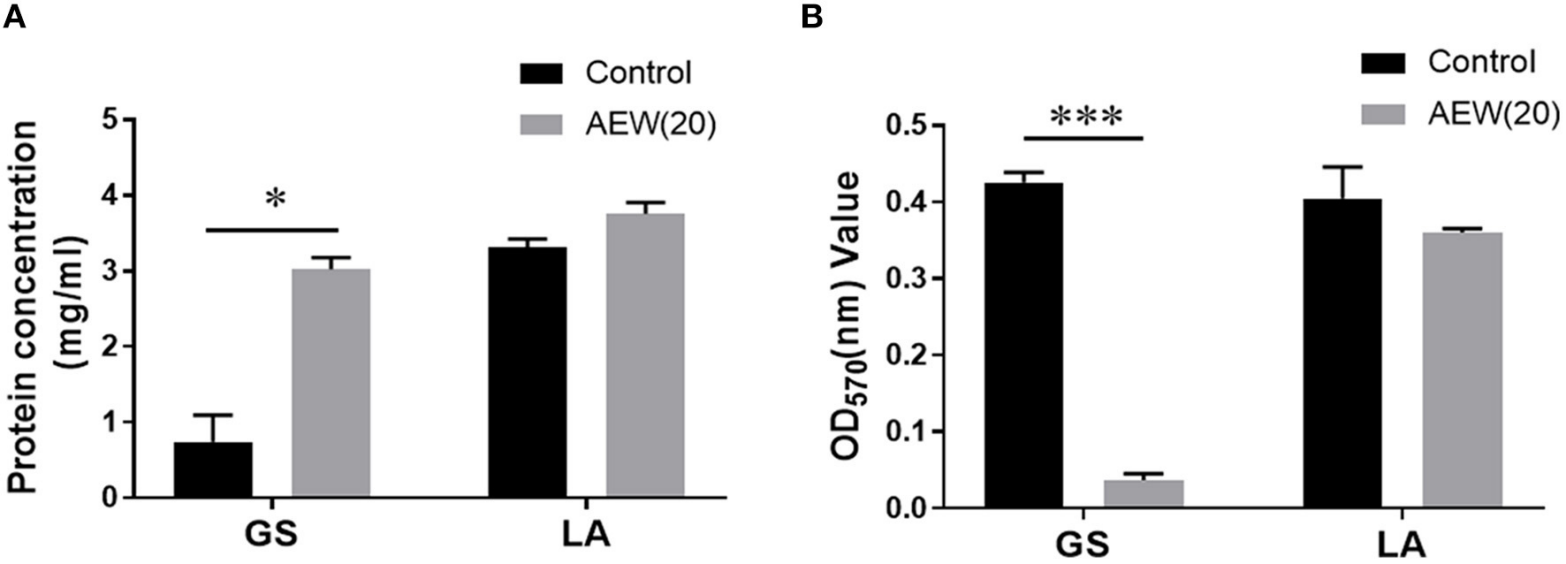
Twenty ppm AEW promoted protein leakage and cell lysis of GS with less effect on LA. **(A)** Statistics showed the GS and LA proteins in the supernatant after 20 ppm AEW treatment using the BCA method (n = 3, *p < 0.05 vs. control). **(B)** The data showed the absorbance of GS and LA precipitates after 20 ppm AEW treatment (*n* = 3, ***p < 0.001 vs. control).

To further delineate the destructive effect of AEW (20 ppm) on cell structure, we applied BCA protein determination and OD value determination technology to evaluate the cell leakage protein and bacterial lysis after AEW treatment. The results demonstrated that protein in the supernatant of GS group increased from (0.74 ± 0.29 mg/ml) to (3.02 ± 0.12 mg/ml) after AEW treatment (Figure 3A, *n* = 3, vs. control, ^*^p < 0.05). It was worth noting that there was no significant change in the supernatant protein of the LA group after AEW treatment (3.75 ± 0.12 mg/ml) (Figure 3A, *n* = 3, vs. control, *p* > 0.05).

Additionally, after removing the treatment solution, the absorbance of the GS treatment group (0.03 ± 0.01) was much less than that of the control group (0.43 ± 0.01) (Figure 3B, *n* = 3, ^*^p < 0.05). However, for LA, there was no significant difference between the treatment group (0.40 ± 0.03) and the control group (0.42 ± 0.01) (Figure 3B, *n* = 3, *p* > 0.05).

### Twenty ppm AEW Induced ROS Production in GS Whereas It Has Less Effect on LA

**Figure 4:**
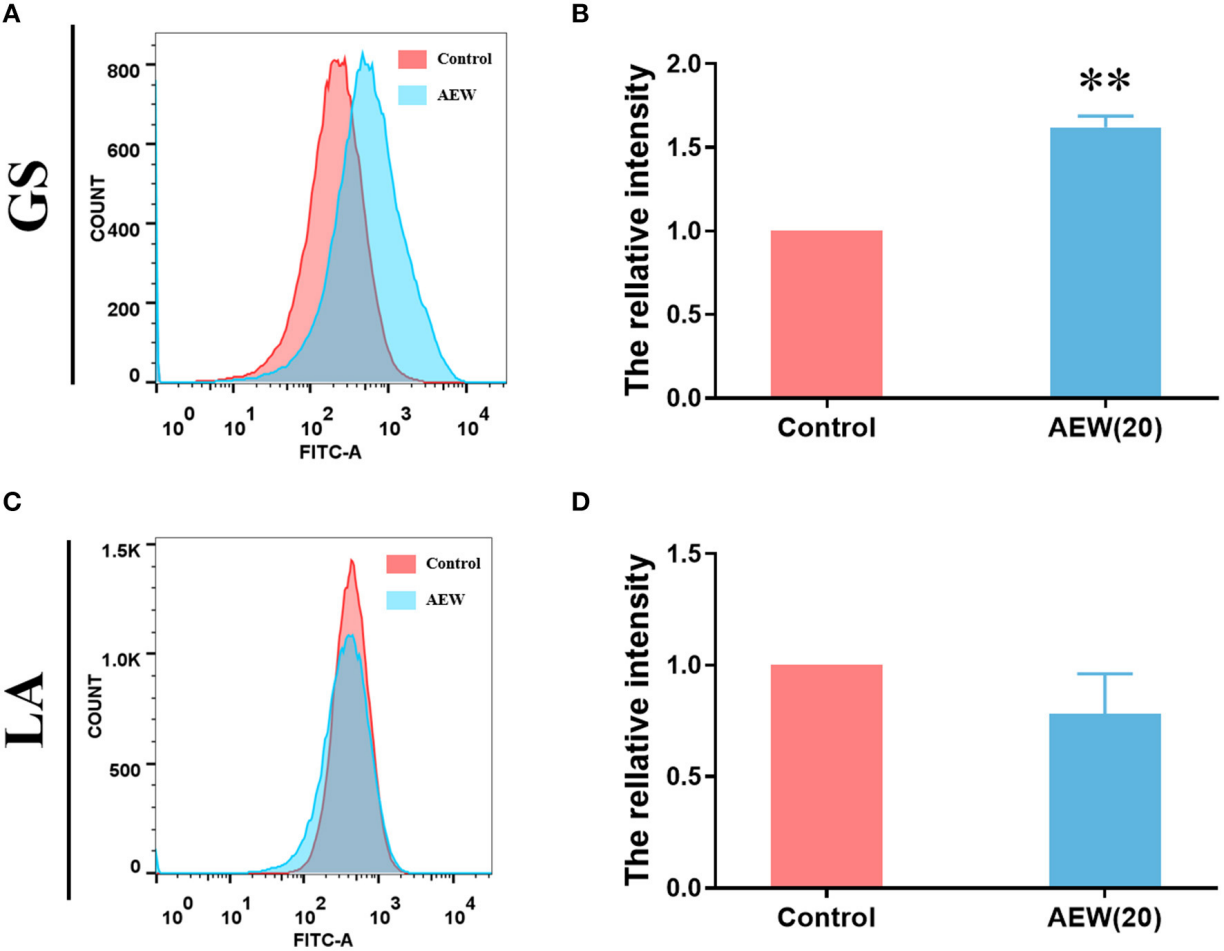
Twenty ppm AEW induced ROS production in GS whereas it had less effect on LA. **(A,C)** Representative DCF fluorescence data were observed by flow cytometry. **(B,D)** Statistics of relative DCF fluorescence intensity of GS and LA were measured by flow cytometry (*n* = 3, **p < 0.01 to control).

Recent studies have shown that AEW can also regulate intracellular ROS metabolism (29). Under flow cytometry, we detected that the relative average fluorescence intensity of GS treated with AEW was markedly higher than that of the control group (Figures 4A,B, *n* = 3, ^*^p < 0.05). However, the relative average fluorescence intensity of the AEW treatment group was not memorably different from that of the control group in LA (Figures 4C,D, *n* = 3, *p* > 0.05). The ROS qualitative experiment also demonstrated that after 20 ppm AEW treatment, the ROS of GS was produced in large quantities (Supplementary Figure S5).

### Twenty ppm AEW Significantly Inhibited the Microbial Viability of Vaginal Secretions of BV Patients

**Figure 5:**
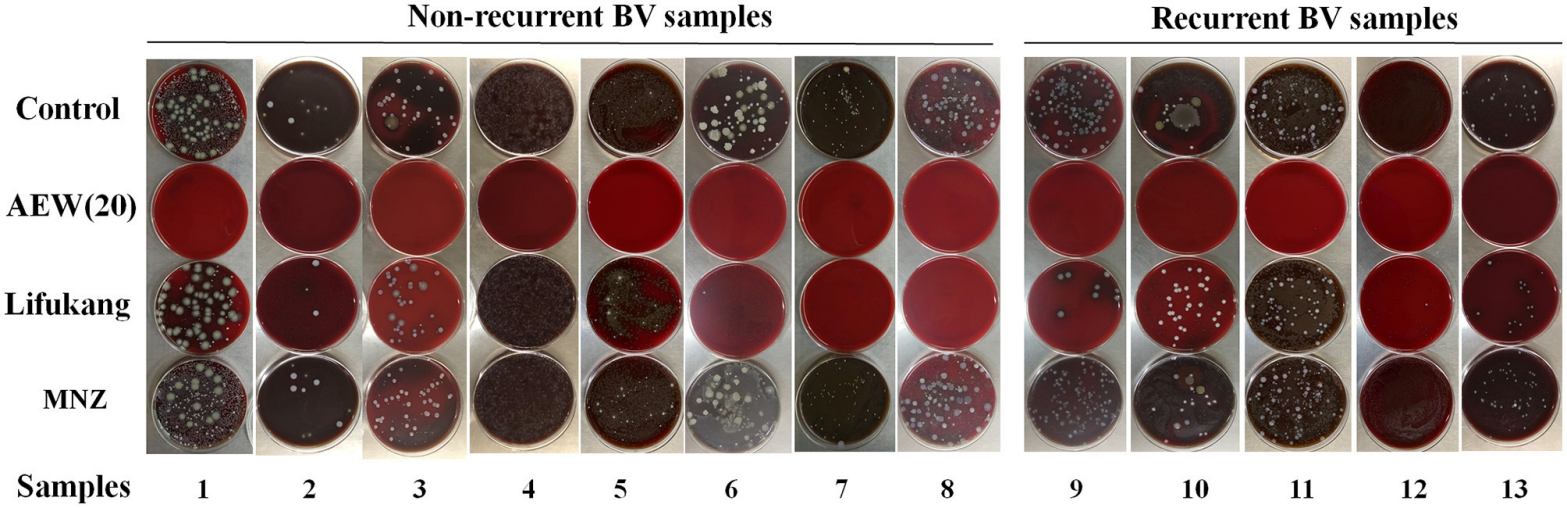
Twenty ppm AEW significantly inhibited the microbial viability of vaginal secretions of BV patients. The picture showed the bacterial growth of the vaginal secretions of BV patients after 20 ppm AEW, Lifukang, and MNZ treatments.

To further explore the potential clinical application of AEW, we collected vaginal secretions from 13 BV patients (diagnosis was based on Amsel criteria and sialic acid plum method) and performed a qualitative analysis of the antivaginal microbial effect of AEW. BV patients included 5 relapsed patients and 8 non-relapsed patients (Figure 5). BV recurrence is a clinically tricky problem. Metronidazole will still recur after a period of treatment, which may be related to the resistance of BV-related bacteria that cannot be eliminated (30).

We found that 20 ppm AEW was better in inhibiting microbial viability in vaginal secretions in both relapsed and non-relapsed BV samples. In contrast, Lifukang was less effective against BV patient secretion microbes than 20 ppm AEW. Some plates (plates 1–6, 9–13) still had bacterial growth. In addition, Lifukang was less effective against relapsed patient samples than non-relapsed samples (bacterial growth on all plates, 9–13). MNZ was the least effective at inhibiting microbial viability in vaginal secretions (bacterial growth on all plates, 1–13) (Figure 5).

## Discussion

Bacterial vaginosis is a dysbiosis caused by vaginal microbiota imbalance. Excessive growth of pathogenic bacteria will increase the production of volatile amines and increase the vaginal pH to >4.5 (31). Increased vaginal pH will not only aggravate the progression of BV and increase the susceptibility to BV but also be related to premature delivery (32). Accordingly, it is vital to promote the recovery of the acidic environment of the vagina when treating BV (33). Some new drugs had significant antibacterial effects in treating vaginal diseases (34). Nevertheless, these new drugs face the same dilemma as MNZ (pH 5.67) and Lifukang (pH 5.29), which have a slightly higher pH. What is worthy of our attention is that AEW has the characteristics of adjustable pH. In recent years, various electrolyzed aquatic products have been promoted and used, such as weakly alkaline, strong acid, neutral, weak acid, and other AEW (35). Based on this advantage, we first adjusted the pH value of AEW (3.71–4.22), which is close to the normal vaginal pH value (3.8–4.5). The decrease in pH is conducive to the survival and recolonization of LA (36). Plate counting experiment illustrated that AEW ≥20 ppm could significantly inhibit the culturability of GS, but showed a negligible effect on LA. These results proved that AEW (20 ppm) not only has acidic properties but also inhibits GS growth well. Further exploration of low concentrations of AEW is needed to determine the minimum inhibitory concentration, which will help us screen out the most ideal AEW concentration. Of course, the problems must be faced that the fine-tuning of AEW is challenging to achieve at this moment, such as the irregularity of dilution concentration (37), the stability of water pressure (38), and so on. These are all to be resolved.

Second, we preliminarily explored the possible antibacterial mechanisms behind the different inactivation effects of 20 ppm AEW on GS and LA. Scientists have discovered that the antibacterial mechanism of AEW may be related to the destruction of the integrity of cell membranes (39). In this study, the Hoechst staining and PI staining of GS increased significantly by AEW treatment, which may be caused by the destruction of the integrity of the cell membrane and the infiltration of the stain. The increase in PI staining is not only the result of cell necrosis but may also be a manifestation of apoptosis. Our previous studies have confirmed that AEW can promote cell apoptosis in *Escherichia coli* (22). Then, we investigated the specific manifestations of this integrity breach using transmission electron microscopes (TEMs). TEM results exhibited that the GS cell membrane was wrinkled, and cytoplasm began to show vacuoles and low density after AEW treatment. Kim et al. also observed that after AEW treatment, the cell wall had round holes through which the cytoplasmic content was discharged (23). In fact, a previous study showed that superoxidized water might destroy the covalent bonds of proteins, causing them to break, which might cause the proteins to shrink and flow out of the damaged cell membrane (40). In this study, we found that after AEW treatment, a large amount of protein leaked in GS, and the precipitation of GS cells reduced. This may be due to the fragmentation of GS cells or the drastic reduction of cell content, which causes GS to be discarded during the centrifugation process.

The abovementioned 20 ppm AEW caused cell damage almost did not occur on LA. AEW is prone to oxidative stress in cells due to its high oxidation potential (41). Studies have found that the polysaccharides on LA's surface may promote its antioxidant capacity (42). In our study, 20 ppm AEW induced the ROS to increase significantly in GS, whereas this phenomenon could not be observed in LA, which strongly proved that the antioxidant capacity of LA made it resistant to the oxidative stress damage caused by AEW. However, it is not clear which kind of polysaccharides or antioxidant enzymes play critical roles in LA combating the damage caused by AEW. This requires further experiments to verify.

Finally, we observed the antibacterial effect of 20 ppm AEW on the vaginal secretions of women diagnosed with BV by Amsel criteria and sialic acid plum method. To our surprise, 20 ppm AEW was better at inhibiting microbial viability in vaginal secretions than Lifukang and MNZ. We needed to note that some samples (samples 1, 3, 6, and 10) had apparent fungal-like colony growth, which indicated that the samples may have a mixed infection of bacteria and candida. The mixed infection of bacteria and candida is a complex problem to solve clinically, and its proportion is increasing year by year (43). In the clinical treatment of recurrent BV, in addition to the use of MNZ, it is necessary to increase antifungal drug therapy (44). Thus, it can be seen that AEW has a broad spectrum of antimicroorganisms. In addition, AEW has the advantages of simple preparation, lower price, and better safety (45, 46).

In summary, 20 ppm AEW may cause oxidative damage to GS, leading to the destruction of cell integrity, increasing cell permeability and leakage of contents, and ultimately leading to the death of GS. Due to the antibacterial potential, acidity, simple preparation, and cheapness of AEW (20 ppm), it may be used in the treatment of BV in the future. Of course, we still have a lot of work to be done. Although some experiments have proven that AEW >20 ppm with not toxic and irritating to the abdominal cavity, nasal mucosa, and other tissues (47, 48), further experiments are still needed to explore the toxicity and irritation of AEW to vaginal epithelium. The use of neutralizers for AEW also should be explored its irritation and toxicity to the vaginal epithelium. Besides, due to the multibacteria of the vaginal flora in BV status, it is necessary to further test the antibacterial potential of AEW (≤20 ppm) on other Lactobacilli (such as *L. crispatus* and *L. jenseni*) and BV-related bacteria (such as *Atopobium vagae, Mobiluncus* spp., and *Prevotella bivia*), which may involve the future use of AEW to flush to prevent the occurrence of BV.

## Data Availability Statement

The original contributions presented in the study are included in the article/Supplementary Material, further inquiries can be directed to the corresponding authors.

## Ethics Statement

The studies involving human participants were reviewed and approved by Ethics Review Committee of the First Affiliated Hospital of Jinan University. The patients/participants provided their written informed consent to participate in this study.

## Author Contributions

CZ, YC, and LG conducted most of the experiments and wrote the first draft of the manuscript. CZ, YC, JH, XY, and LP analyzed the data. LG collected specimens. LZ and ZY were responsible for experimental design, data analysis and interpretation, writing, revising, and finalization of manuscripts. All authors have read and approved the final manuscript.

## Funding

This work was supported by the National Natural Science Foundation of China (no. 81872133). The authors also declare that Fuqiang Want Sanitary Accessories Ltd provided funding (no. 40118150), electrolyzed water equipment and technology. Fuqiang Want Sanitary Accessories Ltd was not involved in the study design, collection, analysis, interpretation of data, the writing of this article or the decision to submit it for publication.

## Conflict of Interest

The authors declare that the research was conducted in the absence of any commercial or financial relationships that could be construed as a potential conflict of interest.

## Publisher's Note

All claims expressed in this article are solely those of the authors and do not necessarily represent those of their affiliated organizations, or those of the publisher, the editors and the reviewers. Any product that may be evaluated in this article, or claim that may be made by its manufacturer, is not guaranteed or endorsed by the publisher.

## Supplementary Material

Supplementary Figure 1 Schematic diagram of a AEW generator. Anode side:

(1)
$${\text{H}}_{2}\text{O}\to 1/2{\text{O}}_{2}+2{\text{H}}^{+}+2{\text{e}}^{-}$$

(2)
$$2\text{C}{\text{l}}^{-}\to \text{C}{\text{l}}_{2}+2{\text{e}}^{-}$$

(3)
$$\text{C}{\text{l}}_{2}(\text{aq})+{\text{H}}_{2}\text{O}⇆\text{HClO}+\text{HCl}$$

Cathode side:

(4)
$$\begin{array}{l} 2{\text{H}}_{2}\text{O}+2{\text{e}}^{-}\to 2\text{O}{\text{H}}^{-}+{\text{H}}_{2}\left(4\right) \end{array}$$

Supplementary Figure S2 Flowchart for exploring the effects of AEW, MNZ, and Lifukang on microbial inactivation. Flow diagram showing experiments designed to determine sanitization potency of AEW, MNZ, and Lifukang on pure cultures of GS and LA treated for 5 min, 10 min, and 20 min.

Supplementary Figure S3 Comparison of antibacterial rate of 20 ppm AEW, Lifukang, MNZ to LA and GS. The picture showed the antibacterial rate of 20 ppm AEW, Lifukang, and MNZ for LA and GS in different periods.

Supplementary Figure S4 Investigating bacterial staining by PI and Hoechst using fluorescence microscopy after AEW (20 ppm) treatment. Fluorescence labeled with Hoechst (blue) **(A)** and PI (red) **(B)** indicated bacterias with increased cell membrane permeability (indicated by arrows).

Supplementary Figure S5 Exploring ROS production in GS and LA using fluorescence microscopy after AEW (20 ppm) treatment. Representative DCF fluorescence of ROS in GS and LA was observed under a fluorescence microscope after 20 ppm AEW treatment (indicated by arrows).

## References

[1] Onderdonk AB Delaney ML Fichorova RN. The human microbiome during bacterial vaginosis. Clin Microbiol Rev. (2016) 29:223–38. 10.1128/CMR.00075-15 26864580 PMC4786887

[2] Petrova MI Lievens E Malik S Imholz N Lebeer S. Lactobacillus species as biomarkers and agents that can promote various aspects of vaginal health. Front Physiol. (2015) 6:81. 10.3389/fphys.2015.00081 25859220 PMC4373506

[3] Ravel J Gajer P Abdo Z Schneider GM Koenig SS McCulle SL. Vaginal microbiome of reproductive-age women. Proc Natl Acad Sci USA. (2011) 108(Suppl. 1):4680–7. 10.1073/pnas.1002611107 20534435 PMC3063603

[4] Workowski KA Bachmann LH Chan PA Johnston CM Muzny CA Park I. Sexually transmitted infections treatment guidelines, 2021. MMWR Recomm Rep. (2021) 70:1–187. 10.15585/mmwr.rr7004a1 34292926 PMC8344968

[5] Bradshaw CS Vodstrcil LA Hocking JS Law M Pirotta M Garland SM. Recurrence of bacterial vaginosis is significantly associated with posttreatment sexual activities and hormonal contraceptive use. Clin Infect Dis. (2013) 56:777–86. 10.1093/cid/cis1030 23243173

[6] Chavoustie SE Jacobs M Reisman HA Waldbaum AS Levy SF Hillier SL. Metronidazole vaginal gel 1.3% in the treatment of bacterial vaginosis: a dose-ranging study. J Lower Genital Tract Dis. (2015) 19:129–34. 10.1097/LGT.0000000000000062 24983350 PMC4376277

[7] Machado D Castro J Palmeira-de-Oliveira A Martinez-de-Oliveira J Cerca N. Bacterial vaginosis biofilms: challenges to current therapies and emerging solutions. Front Microbiol. (2016) 6:1528. 10.3389/fmicb.2015.01528 26834706 PMC4718981

[8] Muñoz-Barreno A Cabezas-Mera F Tejera E Machado A. Comparative effectiveness of treatments for bacterial vaginosis: A network meta-analysis. Antibiotics. (2021) 10:978. 10.3390/antibiotics10080978 34439028 PMC8388924

[9] Verstraelen H Verhelst R Roelens K Temmerman M. Antiseptics and disinfectants for the treatment of bacterial vaginosis: a systematic review. BMC Infect Dis. (2012) 12:148. 10.1186/1471-2334-12-148 22742642 PMC3458956

[10] Palmeira-de-Oliveira A Silva BM Palmeira-de-Oliveira R Martinez-de-Oliveira J Salgueiro L. Are plant extracts a potential therapeutic approach for genital infections? Curr Med Chem. (2013) 20:2914–28. 10.2174/09298673113209990007 23651308

[11] Bradshaw CS Sobel JD. Current treatment of bacterial vaginosis-limitations and need for innovation. J Infect Dis. (2016) 214(Suppl. 1):S14–20. 10.1093/infdis/jiw159 27449869 PMC4957510

[12] Issa-Zacharia A Kamitani Y Miwa N Muhimbula H Iwasaki K. Application of slightly acidic electrolyzed water as a potential non-thermal food sanitizer for decontamination of fresh ready-to-eat vegetables and sprouts. Food Control. (2011) 22:601–7. 10.1016/j.foodcont.2010.10.011

[13] Cao W Zhu ZW Shi ZX Wang CY Li BM. Efficiency of slightly acidic electrolyzed water for inactivation of *Salmonella enteritidis* and its contaminated shell eggs. Int J Food Microbiol. (2009) 130:88–93. 10.1016/j.ijfoodmicro.2008.12.021 19185376

[14] Guentzel JL Liang Lam K Callan MA Emmons SA Dunham VL. Reduction of bacteria on spinach, lettuce, and surfaces in food service areas using neutral electrolyzed oxidizing water. Food Microbiol. (2008) 25:36–41. 10.1016/j.fm.2007.08.003 17993375

[15] You HS Fadriquela A Sajo MEJ Bajgai J Ara J Kim CS. Wound healing effect of slightly acidic electrolyzed water on cutaneous wounds in hairless mice via immune-redox modulation. Biol Pharm Bull. (2017) 40:1423–31. 10.1248/bpb.b17-00219 28867725

[16] Takeda Y Jamsransuren D Makita Y Kaneko A Matsuda S Ogawa H. Inactivation activities of ozonated water, slightly acidic electrolyzed water and ethanol against SARS-CoV-2. Molecules. (2021) 26:5465. 10.3390/molecules26185465 34576934 PMC8471879

[17] Alonzo Martínez MC Cazorla E Cánovas E Martínez-Blanch JF Chenoll E Climent E. Study of the vaginal microbiota in healthy women of reproductive age. Microorganisms. (2021) 9:1069. 10.3390/microorganisms9051069 34063526 PMC8156707

[18] Ling Z Kong J Liu F Zhu H Chen X Wang Y. Molecular analysis of the diversity of vaginal microbiota associated with bacterial vaginosis. BMC Genomics. (2010) 11:488. 10.1186/1471-2164-11-488 20819230 PMC2996984

[19] Drago L De Vecchi E Nicola L Zucchetti E Gismondo MR Vicariotto F. Activity of a *Lactobacillus acidophilus*-based douche for the treatment of bacterial vaginosis. J Altern Complement Med. (2007) 13:435–8. 10.1089/acm.2006.6040 17532736

[20] Jang SE Jeong JJ Choi SY Kim H Han MJ Kim DH. *Lactobacillus rhamnosus* HN001 and *Lactobacillus acidophilus* La-14 attenuate *Gardnerella vaginalis*-infected bacterial vaginosis in mice. Nutrients. (2017) 9. 10.3390/nu9060531 28545241 PMC5490510

[21] Dean SN Leary DH Sullivan CJ Oh E Walper SA. Isolation and characterization of *Lactobacillus*-derived membrane vesicles. Sci Rep. (2019) 9:877. 10.1038/s41598-018-37120-6 30696852 PMC6351534

[22] Ye Z Wang S Chen T Gao W Zhu S He J. Inactivation mechanism of *Escherichia coli* induced by slightly acidic electrolyzed water. Sci Rep. (2017) 7:6279. 10.1038/s41598-017-06716-9 28740247 PMC5524752

[23] Kim HJ Tango CN Chelliah R Oh DH. sanitization efficacy of slightly acidic electrolyzed water against pure cultures of *Escherichia coli, Salmonella enterica, Typhimurium, Staphylococcus aureus* and *Bacillus cereus* spores, in comparison with different water hardness. Sci Rep. (2019) 9:4348. 10.1038/s41598-019-40846-6 30867518 PMC6416306

[24] Liu X Zhang M Meng X He X Zhao W Liu Y. Inactivation and membrane damage mechanism of slightly acidic electrolyzed water on *Pseudomonas deceptionensis* CM2. Molecules. (2021) 26:1012. 10.3390/molecules26041012 33672940 PMC7917946

[25] Amsel R Totten PA Spiegel CA Chen KC Eschenbach D Holmes KK. nonspecific vaginitis. Diagnostic criteria and microbial and epidemiologic associations. Am J Med. (1983) 74:14–22. 10.1016/0002-9343(83)91112-9 6600371

[26] Anstey Watkins J Ross JDC Thandi S Brittain C Kai J Griffiths F. Acceptability of and treatment preferences for recurrent bacterial vaginosis-Topical lactic acid gel or oral metronidazole antibiotic: qualitative findings from the VITA trial. PLoS ONE. (2019) 14:e0224964. 10.1371/journal.pone.0224964 31730666 PMC6857901

[27] Veasey S Muriana PM. Evaluation of electrolytically-generated hypochlorous acid ('Electrolyzed Water') for sanitation of meat and meat-contact surfaces. Foods. (2016) 5:42. 10.3390/foods5020042 28231137 PMC5302345

[28] Zhang N Fan Y Li C Wang Q Leksawasdi N Li F. Cell permeability and nuclear DNA staining by propidium iodide in basidiomycetous yeasts. Appl Microbiol Biotechnol. (2018) 102:4183–91. 10.1007/s00253-018-8906-8 29572560

[29] Chen Y Hung YC Chen M Lin M Lin H. Enhanced storability of blueberries by acidic electrolyzed oxidizing water application may be mediated by regulating ROS metabolism. Food Chem. (2019) 270:229–35. 10.1016/j.foodchem.2018.07.095 30174039

[30] Gustin AT Thurman AR Chandra N Schifanella L Alcaide M Fichorova R. Recurrent bacterial vaginosis following metronidazole treatment is associated with microbiota richness at diagnosis. Am J Obstet Gynecol. (2021) 226:225.e1–15. 10.1016/j.ajog.2021.09.018 34560047 PMC8887553

[31] Fredricks DN Fiedler TL Marrazzo JM. Molecular identification of bacteria associated with bacterial vaginosis. N Engl J Med. (2005) 353:1899–911. 10.1056/NEJMoa043802 16267321

[32] Aldunate M Srbinovski D Hearps AC Latham CF Ramsland PA Gugasyan R. Antimicrobial and immune modulatory effects of lactic acid and short chain fatty acids produced by vaginal microbiota associated with eubiosis and bacterial vaginosis. Front Physiol. (2015) 6:164. 10.3389/fphys.2015.00164 26082720 PMC4451362

[33] Nyirjesy P. Management of persistent vaginitis. Obstet Gynecol. (2014) 124:1135–46. 10.1097/AOG.0000000000000551 25415165

[34] Algburi A Zhang Y Weeks R Comito N Zehm S Pinto J. Gemini cationic amphiphiles control biofilm formation by bacterial vaginosis pathogens. Antimicrob Agents Chemother. (2017) 61:e00650-17. 10.1128/AAC.00650-17 28893789 PMC5700334

[35] Athayde DR Flores DRM da Silva JS Genro ALG Silva MS Klein B. Application of electrolyzed water for improving pork meat quality. Food Res Int. (2017) 100(Pt 1):757–63. 10.1016/j.foodres.2017.08.009 28873747

[36] Garg KB Ganguli I Kriplani A Lohiya NK Thulkar J Talwar GP. Metabolic properties of lactobacilli in women experiencing recurring episodes of bacterial vaginosis with vaginal pH >or= 5. Eur J Clin Microbiol Infect Dis. (2010) 29:123–5. 10.1007/s10096-009-0818-1 19802749

[37] Lee HW Park B Yoon SR Yang JS Ha JH. Physicochemical stability and virucidal effect of diluted, slightly acidic electrolyzed water against human norovirus. Food Sci Biotechnol. (2022) 31:131–8. 10.1007/s10068-021-01011-w 35059237 PMC8733102

[38] Rahman SME Ding T Oh D-H. Effectiveness of low concentration electrolyzed water to inactivate foodborne pathogens under different environmental conditions. Int J Food Microbiol. (2010) 139:147–53. 10.1016/j.ijfoodmicro.2010.03.020 20385418

[39] Chen TY Kuo SH Chen ST Hwang DF. Differential proteomics to explore the inhibitory effects of acidic, slightly acidic electrolysed water and sodium hypochlorite solution on *Vibrio parahaemolyticus*. Food Chem. (2016) 194:529–37. 10.1016/j.foodchem.2015.08.019 26471589

[40] Zinkevich V Beech IB Tapper R Bogdarina I. The effect of super-oxidized water on *Escherichia coli*. J Hosp Infect. (2000) 46:153–6. 10.1053/jhin.2000.0819 11049710

[41] Liao LB Chen WM Xiao XM. The generation and inactivation mechanism of oxidation–reduction potential of electrolyzed oxidizing water. J Food Eng. (2007) 78:1326–32. 10.1016/j.jfoodeng.2006.01.004

[42] Deepak V Ramachandran S Balahmar RM Pandian SRK Sivasubramaniam SD Nellaiah H. *In vitro* evaluation of anticancer properties of exopolysaccharides from *Lactobacillus acidophilus* in colon cancer cell lines. In Vitro Cell Dev Biol Anim. (2016) 52:163–73. 10.1007/s11626-015-9970-3 26659393

[43] Wang H Huang Z Wu Z Qi X Lin D. An epidemiological study on vaginitis in 6,150 women of reproductive age in Shanghai. New Microbiol. (2017) 40:113–8. 28255605

[44] Benyas D Sobel JD. Mixed vaginitis due to bacterial vaginosis and candidiasis. J Low Genit Tract Dis. (2022) 26:68–70. 10.1097/LGT.0000000000000641 34840242

[45] Zhang C Cao W Hung YC Li BJFC. Disinfection effect of slightly acidic electrolyzed water on celery and cilantro. Food Control. (2016) 69:147–52. 10.1016/j.foodcont.2016.04.039

[46] Zhang J Yang H Chan JZY. Development of portable flow-through electrochemical sanitizing unit to generate near neutral electrolyzed water. J Food Sci. (2018) 83:780–90. 10.1111/1750-3841.14080 29469931

[47] Kubota A Goda T Tsuru T Yonekura T Yagi M Kawahara H. Efficacy and safety of strong acid electrolyzed water for peritoneal lavage to prevent surgical site infection in patients with perforated appendicitis. Surg Today. (2015) 45:876–9. 10.1007/s00595-014-1050-x 25387655

[48] Giarratana N Rajan B Kamala K Mendenhall M Reiner G. A sprayable Acid-Oxidizing solution containing hypochlorous acid (AOS2020) efficiently and safely inactivates SARS-Cov-2: a new potential solution for upper respiratory tract hygiene. Eur Arch Otorhinolaryngol. (2021) 278:3099–103. 10.1007/s00405-021-06644-5 33575830 PMC7877500

