# Supplementary figures and images for: Acidic Electrolyzed Water Inhibits the Viability of Gardnerella spp. via Oxidative Stress Response
Source: Front Med (Lausanne). 2022 Feb 25;9:817957. doi: 10.3389/fmed.2022.817957 (PMC8916223; doi:10.3389/fmed.2022.817957)

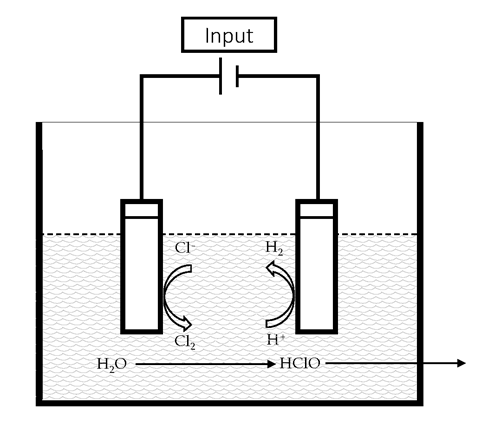

Supplement: Supplementary Figure 1 — Schematic diagram of a AEW generator. [file Image_1.TIF]

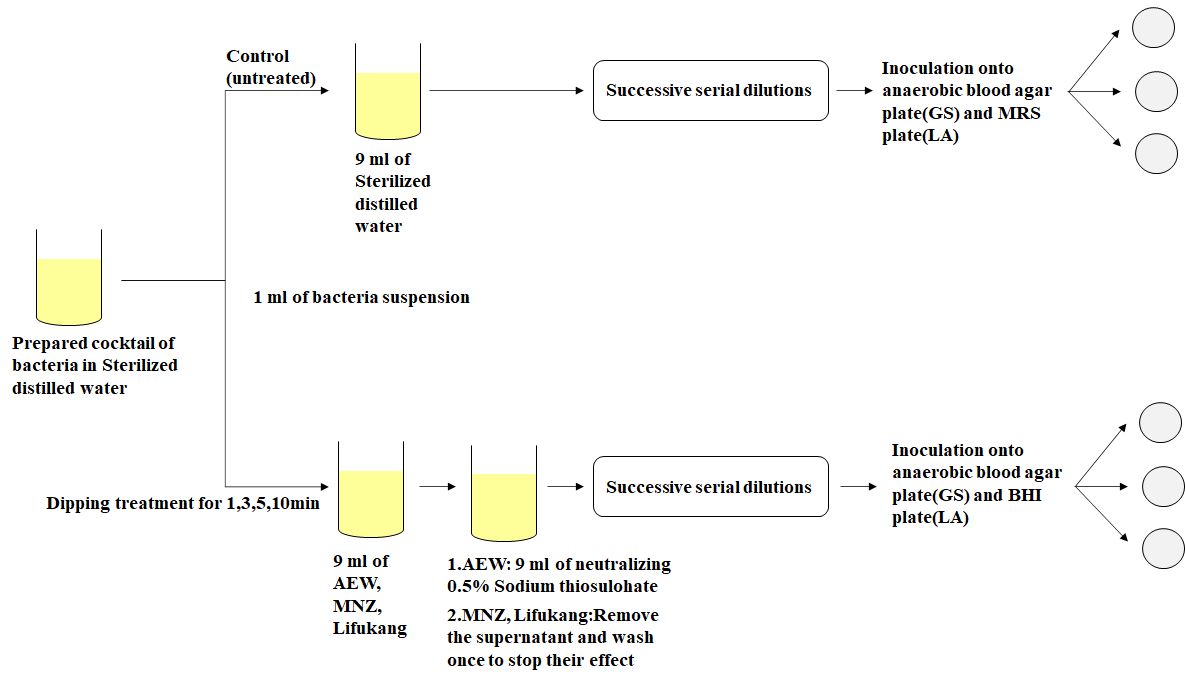

Supplement: Supplementary Figure S2 — Flowchart for exploring the effects of AEW, MNZ, and Lifukang on microbial inactivation. Flow diagram showing experiments designed to determine sanitization potency of AEW, MNZ, and Lifukang on pure cultures of GS and LA treated for 5 min, 10 min, and 20 min. [file Image_2.TIF]

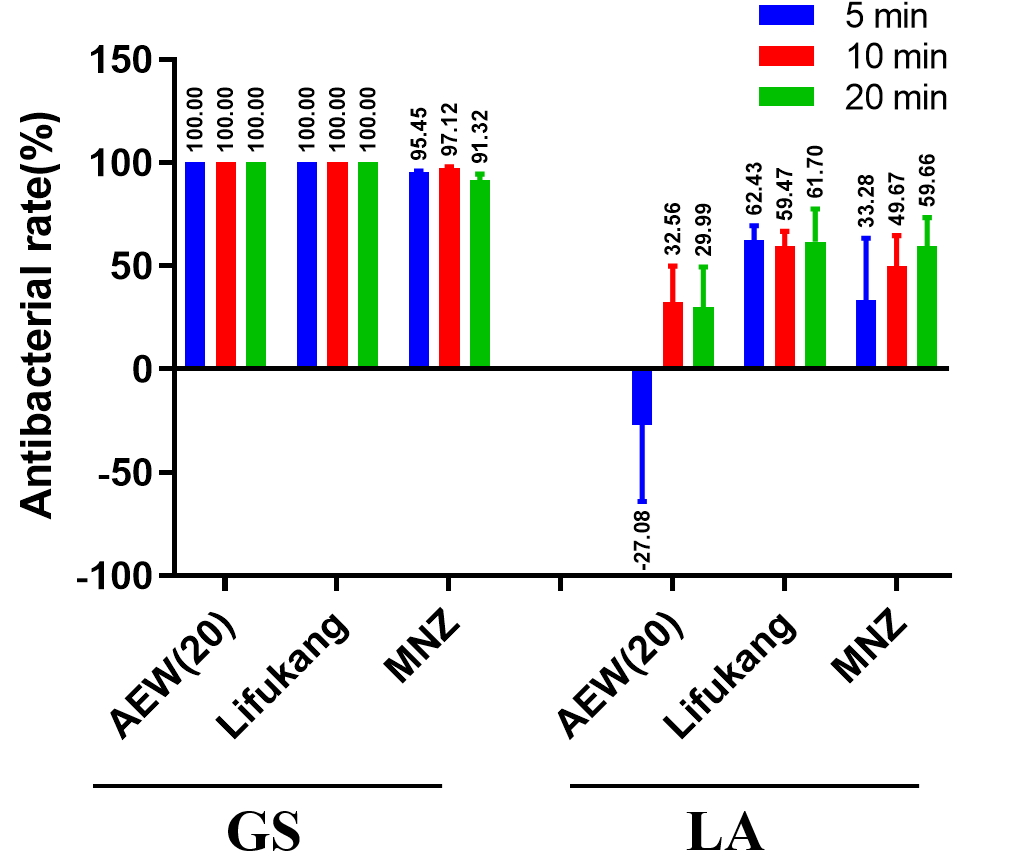

Supplement: Supplementary Figure S3 — Comparison of antibacterial rate of 20 ppm AEW, Lifukang, MNZ to LA and GS. The picture showed the antibacterial rate of 20 ppm AEW, Lifukang, and MNZ for LA and GS in different periods. [file Image_3.TIF]

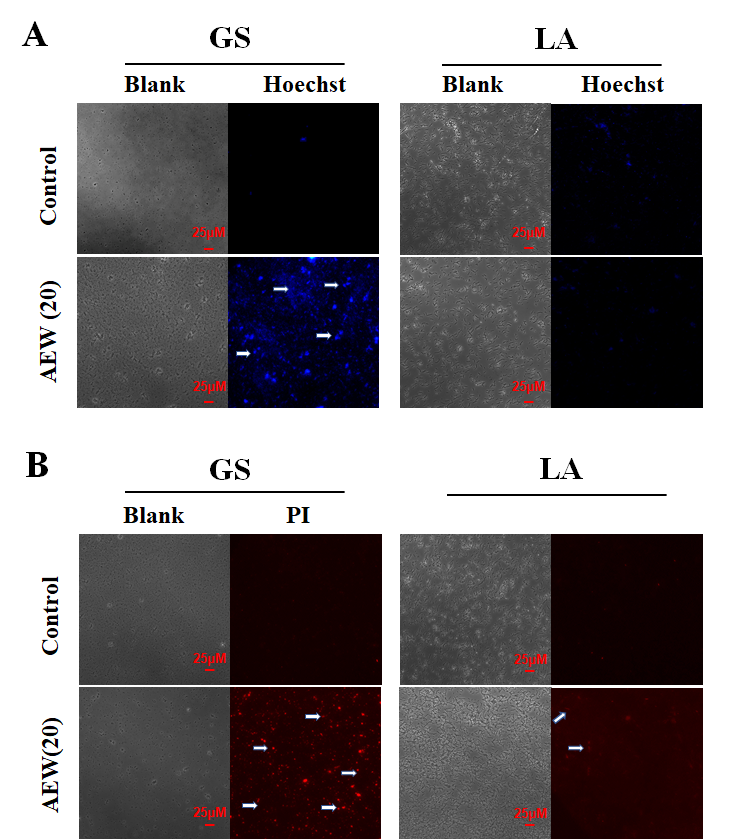

Supplement: Supplementary Figure S4 — Investigating bacterial staining by PI and Hoechst using fluorescence microscopy after AEW (20 ppm) treatment. Fluorescence labeled with Hoechst (blue) (A) and PI (red) (B) indicated bacterias with increased cell membrane permeability (indicated by arrows). [file Image_4.TIF]

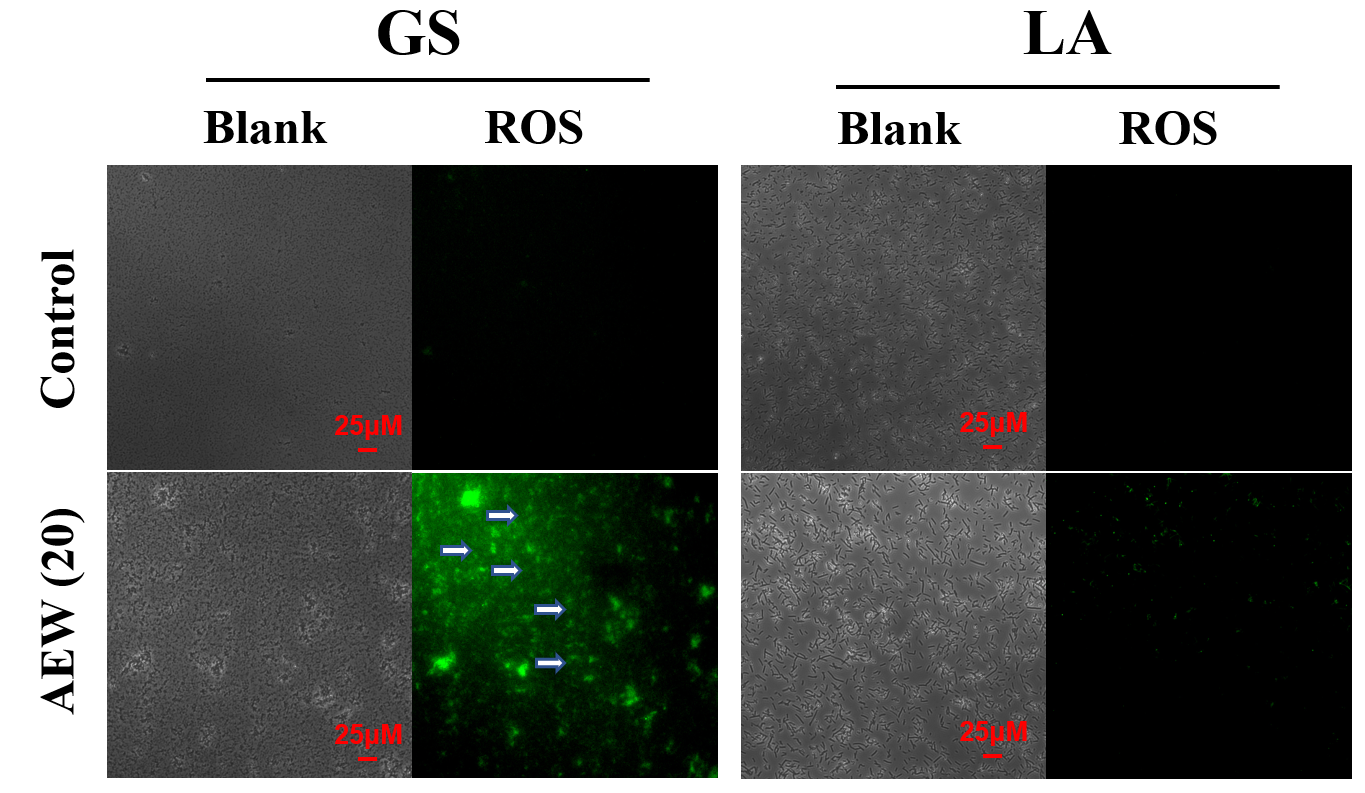

Supplement: Supplementary Figure S5 — Exploring ROS production in GS and LA using fluorescence microscopy after AEW (20 ppm) treatment. Representative DCF fluorescence of ROS in GS and LA was observed under a fluorescence microscope after 20 ppm AEW treatment (indicated by arrows). [file Image_5.TIF]
